# Unlocking antagonistic potential of *Bacillus amyloliquefaciens* KRS005 to control gray mold

**DOI:** 10.3389/fmicb.2023.1189354

**Published:** 2023-06-02

**Authors:** Hong-Yue Qi, Dan Wang, Dongfei Han, Jian Song, Muhammad Ali, Xiao-Feng Dai, Xiao-Jun Zhang, Jie-Yin Chen

**Affiliations:** ^1^College of Life Science and Technology, Mudanjiang Normal University, Mudanjiang, China; ^2^The State Key Laboratory for Biology of Plant Diseases and Insect Pests, Institute of Plant Protection, Chinese Academy of Agricultural Sciences, Beijing, China; ^3^School of Environmental Science and Engineering, Suzhou University of Science and Technology, Suzhou, China; ^4^Sustainable Development Study Centre, Government College University, Lahore, Pakistan; ^5^Western Agricultural Research Center, Chinese Academy of Agricultural Sciences, Changji, China

**Keywords:** gray mold, *Botrytis cinerea*, biological control, *Bacillus amyloliquefaciens*, plant immunity

## Abstract

To establish a safe, efficient, and simple biocontrol measure for gray mold disease caused by *Botrytis cinerea*, the basic characteristics and antifungal activity of KRS005 were studied from multiple aspects including morphological observation, multilocus sequence analysis and typing (MLSA–MLST), physical-biochemical assays, broad-spectrum inhibitory activities, control efficiency of gray mold, and determination of plant immunity. The strain KRS005, identified as *Bacillus amyloliquefaciens*, demonstrated broad-spectrum inhibitory activities against various pathogenic fungi by dual confrontation culture assays, of which the inhibition rate of *B. cinerea* was up to 90.3%. Notably, through the evaluation of control efficiency, it was found that KRS005 fermentation broth could effectively control the occurrence of tobacco leaves gray mold by determining the lesion diameter and biomass of *B. cinerea* on tobacco leaves still had a high control effect after dilution of 100 folds. Meanwhile, KRS005 fermentation broth had no impact on the mesophyll tissue of tobacco leaves. Further studies showed that plant defense-related genes involved in reactive oxygen species (ROS), salicylic acid (SA), and jasmonic acid (JA)-related signal pathways were significantly upregulated when tobacco leaves were sprayed with KRS005 cell-free supernatant. In addition, KRS005 could inhibit cell membrane damage and increase the permeability of *B. cinerea*. Overall, KRS005, as a promising biocontrol agent, would likely serve as an alternative to chemical fungicides to control gray mold.

## 1. Introduction

Gray mold of plants is difficult to control because the pathogen, *Botrytis cinerea*, can endure for a long time as mycelium and/or conidia. *Botrytis cinerea* causes significant losses in a variety of crops worldwide, including tobacco plants. Tobacco gray mold disease occurs during seedling growth and foliar maturity. *Botrytis cinerea* is more destructive on tobacco seedlings, stem bases, and leaves. Gray mold was first found in Japan in 1981 on tobacco and has been found in other countries around the world. The disease was first reported in Guizhou, China, and appeared on the leaves as small spots increased in size and developed into expanded, dark brown lesions under cool, humid conditions (Wang et al., 2011). China is the largest tobacco market, and gray mold occurs in both field and greenhouse crops, reaching more than 40% on many crops if no control is applied (Pedras et al., 2011; Villa-Rojas et al., 2012). Spraying chemical fungicides and biological agents is the control technique for gray mold prevention, the former is the main control method showing high effectiveness in suppressing *B. cinerea*. However, chemical fungicides have a high incidence of resistance that is unfavorable to biocontrol. There are five recognized classes of chemical fungicides based on their mechanisms, ranging from affecting respiration, microtubule assembly, osmotic pressure regulation, and sterol biosynthesis inhibitors to amino acid reversible toxicity (Rosslenbroich and Stuebler, 2000). Chemical reagents were reported to be the most effective reagents in controlling gray mold in agricultural production. However, due to long-time and continuous exposure to the same chemical fungicides, *B. cinerea* has developed significant drug resistance (Leroux et al., 2004). In addition, long-term and large-scale applications of synthetic chemical pesticides would cause serious environmental pollution, disrupt the ecological balance, and threaten the health of humans and animals because of crop residues (Hernández et al., 2013; Bernardes et al., 2015).

Biological control, as its merits in reducing disease occurrence, improvement of plant defense response, and promoting plant growth, has received increased attention in recent years (Nalini and Parthasarathi, 2014; Grzegorczyk and Cirvilleri, 2017; Sun et al., 2022). Furthermore, the biological control method is environment-friendly and safe for humans and animals, which provides long-time prevention of plant diseases through the antagonistic microorganism colonization in soil and rhizosphere (Wan et al., 2008; Wu et al., 2009). In short, due to the advantages of low toxicity and pollution, safety, and efficiency, biocontrol technology has been intensively applied to field control in recent years with remarkable effects (Kaewchai et al., 2009).

Biological control microorganisms (namely antagonistic microorganisms) involving bacteria, fungi, and actinomycetes have been identified and studied as potential biological agents (Fontana et al., 2021; Bonaterra et al., 2022; Pandit et al., 2022). Various biological agents have obvious inhibitory effects on *B. cinerea*, of which an approach based on the genus *Bacillus* has been developed and applied to control plant diseases (Yuan et al., 2017; Agarbati et al., 2022). For instance, *B. amyloliquefaciens* GJ1, *B. subtilis* PTS-394, Z-14, and Pnf-4 have been reported to promote plant growth and enhance resistance to gray mold (Qiao et al., 2017; Chen et al., 2019; Nan et al., 2021). *Bacillus licheniformis* GL174 and MG-4 could inhibit the growth of fungal pathogens and reduce the severity of fungal infections *in vitro* (Nigris et al., 2018; Chen et al., 2019). The antifungal experiments have shown that *Pseudomonas* strain QBA5 can inhibit conidial germination, germ tube elongation, and mycelial growth, which suggested that strain QBA5 has a significant preventive effect against the gray mold (Gao et al., 2018).

Since purified *Bacillus* strains have been shown to inhibit other pathogenic fungi, they are more commonly used in biological control (Ji et al., 2021), of which the antagonistic and growth-promoting functions of *B. amyloliquefaciens* strains were reported in many studies. It has been proved that the active antifungal substance produced by *B. amyloliquefaciens* BA-26 inhibited the mycelial growth of *B. cinerea*, disrupted the cell membrane, and inhibited spore germination (Liu et al., 2019). The culture filtrates and extracts of *B. amyloliquefaciens* RS-25 showed strong cellulase and protease activities and were also inhibitory to the gray mold of grapes (Chen et al., 2019). *Bacillus amyloliquefaciens* NCPSJ7 reduced postharvest grape incidence, spot diameter, and rot index and partially inhibited gray mold (Zhou et al., 2020). Plants infected by pathogens usually exhibit physiological and biochemical changes such as defense responses (Thomma et al., 2001; Díaz et al., 2002). For example, the novel protein inducer of *B. amyloliquefaciens* NC6 causes well-defined hypersensitivity response (HR) necrosis in tobacco and may also trigger the expression of plant defense-related genes involved in salicylic acid (SA), phenylalanine aminolase lyase (PAL), and jasmonic acid (JA) signal pathways (Wang et al., 2016).

In this study, the strain KRS005 with biological control effects was isolated from cotton samples and identified using physiological-biochemical and molecular methods. Then the antagonistic activity against various fungal strains and their ability to induce systemic resistance was confirmed. Further results on the effect of gray mold proved that KRS005 provides a potential for developing and applying biological agents to control plant gray mold.

## 2. Materials and methods

### 2.1. Growth of microbes and plant material

The strain KRS005 was isolated from cotton root tissue. The method of isolation was as follows: the healthy cotton roots were cut into small pieces (about 10 mm) and surface disinfected with 70% ethanol for 1 min, and 1.0% NaClO solution for 5 min. The root samples rinsed with sterilized water were ground in a sterilized mortar to obtain a cell suspension. The resulting cell suspension was diluted for 10^−6^ and striped on Luria-Bertani (LB) medium without antibiotics, followed by incubation at 28°C for 2 days. Single colonies with different cultural traits were picked from the plate and further purified on LB plates to obtain a pure culture of bacteria, respectively.

Plant pathogenic fungi were cultured on potato dextrose agar plates (PDA: glucose, 20 g/L; potato, 200 g/L; agar, 15–20 g/L; ddH_2_O, 1,000 mL) at 25°C. The strain KRS005 was inoculated in Luria-Bertani (LB: yeast extract, 5 g/L; peptone, 10 g/L; NaCl, 10 g/L; agar, 15–20 g/L; ddH_2_O, 1,000 mL). The KRS005 fermentation broth was cultured in a shaking incubator at 200 rpm and 28°C. To improve the antifungal activity of KRS005, the formulation of the medium was optimized according to a previous report (Apine and Jadhav, 2011), including 98% (w/w) of potassium humate, 90% (w/w) of compound amino acid 1.5 g/L, defoaming agents 1 mL, ddH_2_O, 1,000 mL, which named as LB-OP. The cell-free supernatant of KRS005 was prepared as the following method: the strain KRS005 was inoculated in a 500 mL flask containing 300 mL optimized LB-OP broth at 28°C and shaking at 200 rpm for 4 days. Then, the fermentation supernatant was harvested by centrifugation at 10,000 rpm. The generated supernatant was filtered using a 0.22 μm Millipore filter to obtain the cell-free supernatant.

Tobacco seedlings (*Nicotiana benthamiana* LAB) were cultivated in a greenhouse with 16 h-light/8 h-dark photoperiods at 25°C for 4 weeks. Each experiment was conducted with the same batch of tobacco seedlings.

### 2.2. Identification of physiological and biochemical characteristics

To detect the characteristics of KRS005, the isolate was streaked on an LB plate. Cells from a single colony were used for gram staining according to Moyes et al. (2009). For detecting phosphate solubilization ability, KRS005 was cultured on the Pikovskava (PVK) medium [glucose 10.0 g/L, (NH_4_)_2_SO_4_ 0.5 g/L NaCl 0.3 g/L MgSO_4_ 0.3 g/L, MnSO_4_ 0.03 g/L, KCl 0.3 g/L, FeSO_4_ 0.03 g/L, Ca_3_(PO_4_)_2_ 5.0 g/L, agar 15.0 g/L, ddH_2_O 1,000 mL pH 7.4], followed by incubation at 28°C for 2–3 days. For the observation of nitrogen fixation ability, the KRS005 was cultured on the nitrogen-free agar medium (KH_2_PO_4_ 0.2 g/L, NaCl 0.2 g/L, MgSO_4_ 0.2 g/L, CaCO_3_ 5.0 g/L, K_2_SO_4_ 0.1 g/L, glucose 10.0 g/L, agar 15.0 g/L, ddH_2_O 1,000 mL pH 7.4) at 28°C for 2–3 days. In addition, Alexandrov medium (sucrose 5.0 g/L, MgSO_4_·7H_2_O 0.5 g/L, CaCO_3_ 0.1 g/L, Na_2_HPO_4_ 2.0 g/L, FeCl_3_·6H_2_O 0.005 g/L, glass powder/ potash feldspar, 1.0 g/L, agar 15 g/L, ddH_2_O 1,000 mL pH 7.4) was used to determine the potassium solubilization ability. Siderophore production of KRS005 was determined according to the description of Schwyn and Neilands (1987) and Wang et al. (2023). Protease activity assay was performed with the medium prepared with 10 mL fresh skim milk, yeast 0.5 g/L, extract 0.5 g/L, NaCl 15 g/L, ddH_2_O 1,000 mL, pH 7.2. The inorganic salt medium [K_2_HPO_4_ 1.0 g/L, MgSO_4_ 1.0 g/L, NaCl 1.0 g/L, (NH_4_)_2_SO_4_ 2.0 g/L, CaCO_3_ 2.0 g/L, FeSO_4_ 0.001 g/L, MnCl_2_ 0.001 g/L, ZnSO_4_ 0.001 g/L, soluble starch 10.0 g/L, ddH_2_O 1,000 mL, pH 7.2] was used to determine the amylase activity. The medium for the measurement of citrate utilization activity included NaCl 5.0 g/L, MgSO_4_·7H_2_O 0.2 g/L, (NH_4_)_2_HPO_4_ 1 g/L, K_2_HPO_4_ 1 g/L, sodium citrate 2 g/L, ddH_2_O 1,000 mL, adjusting pH to 7.0 with 10 mL of 1% bromophenol blue.

In all of the physiological and biochemical tests, *Escherichia coli* DH5α was used as a control. Each treatment was conducted in three replicates, and every test was performed at least three times. All plates were cultured for 24 h at 28°C.

### 2.3. Phylogenetic analysis

The amplification of *gyrB*, *gyrA,* and *rpoB* genes was performed through the following process: the initial degeneration at 95°C for 3 min, followed by 33 cycles at 95°C for 15 s, 60°C for 20 s, 72°C for 2 min, and finally 72°C for 5 min. The PCR products were sequenced and aligned, respectively, in the NCBI database.^1^ The phylogenetic tree was constructed with MEGA6 using maximum likelihood. The bootstrap of 10,000 replications was performed to assess the relative stability of the branches. The used primer pairs were listed in Supplementary Table S1.

### 2.4. Determination for antifungal activity of KRS005 *in vitro*

In total, six plant pathogenic fungi were treated with stain KRS005 to assess the broad-spectrum inhibitory activities. A measure of 20 μL KRS005 fermentation broth was streaked 20 mm away from the plate center, where a pathogenic mycelial disc of 5 mm was placed in the center of the PDA plate.

The pathogenic bacteria semi-diameter of the KRS005 treatment side and control side were denoted as “A” and “B,” respectively. And the inhibition rate (IR) was calculated as the formula: IR (%) = [(B-A)/B] × 100. All of the pathogenic fungi were treated with three different concentrations (5, 10, and 15% (v/v), respectively) in the PDA plate, and the untreated PDA plate was used for the control. All of the plates were incubated at 25°C for 3–7 days, the morphology of each treatment was observed, and the distance from the center of the colony to its edge was measured. The pathogenic bacteria diameter of the KRS005 treatment side and control side were denoted as “C” and “D,” respectively. And the inhibition rate (IR) was calculated as the formula: IR (%) = [(D − C)/D] × 100. Three replicates were maintained for each treatment, and the assay was conducted twice.

### 2.5. Control effect of KRS005 against gray mold of tobacco

In order to evaluate the biocontrol activity of isolate KRS005 against gray mold caused by *B. cinerea*, four-week-old tobacco seedlings were treated with KRS005 fermentation broth (1.8 × 10^8^ CFU/mL), fragmentized liquid (FL), and cell-free supernatant, respectively, by spraying on the tobacco leaves. The treatment of spraying LB broth on leaves was set as a control. After 24 h, the plant was inoculated with *B. cinerea* strain. The treatment plants were placed in the incubator with 25°C and 80% humidity for 4 days. The diameter of the lesion in leaves under different treatments was measured, and the biomass was detected by quantitative PCR (qPCR). The different treatments were performed on at least six leaves, and the experiment was repeated three times independently.

### 2.6. Analysis of relative fungal biomass

Genomic DNA of *B. cinerea* was extracted using a DNA isolation mini kit (TransGen, Beijing, China). In fungal biomass analysis, qPCR was performed with tobacco elongation factor *NbEF1-α* as internal reference genes to quantify the DNA of *B. cinerea* using the target of *BcActin*. The amplification reaction was conducted using 2 × TransStart Top Green qPCR SuperMix (TransGen Biotech, Beijing, China) and the QuantStudio 5 Real-Time PCR system (Thermo Fisher Scientific, United States). The cycling parameters included initial denaturation at 95°C for 3 min, followed by 40 cycles of 95°C denaturation for 15 s, 60°C annealing for 20 s, and 72°C extension for 20 s. The 2^−ΔΔCT^ method (Livak and Schmittgen, 2001) based on CT values was used to calculate the relative expression level. The qPCR experiment was repeated twice, and each treatment contained three technical replicates. All the primer pairs are listed in Supplementary Table S1.

### 2.7. Detection of plant defense-related genes expression

Four-week-old tobacco leaves were treated with KRS005 fermentation broth, and the ddH_2_O treatment was used as a control. The spraying plants were cultivated at 25°C under a 16 h light/ 8 h dark greenhouse. The tobacco mesophyll cells were observed at 24 h. The tobacco leaves were injected with different cell-free supernatant concentrations [5, 10, 15, 20, 25, 30, 35, and 40% (v/v)]. LB-OP, GFP, and VdEG1 were used as negative and positive controls, respectively. The VdEG1 acted as a pathogen-associated molecular pattern (PAMP) to trigger plant immunity response and induce cell death (Gui et al., 2017). Reactive oxygen species (ROS) burst was detected with a 3′3-diaminobenzidine (DAB) solution for 8 h, as described previously (Bindschedler et al., 2006). The leaves were heated for 20 min in ethanol to eliminate chlorophyll. The optical density value is directly related to the mass of the stain. The more the substance is being measured, the higher the optical density value (Varghese et al., 2014). The results were photographed and observed with a stereomicroscope. The optical density was calculated using ImageJ software.

The defense response-related gene expression levels were detected using reverse transcription-quantitative PCR (RT-qPCR). The leaf samples were collected at 6 h for RNA extraction (TransGen, Beijing, China). Firstly, first-strand cDNA was synthesized from 400 ng of total RNA using TransScript II One-Step gDNA Removal and cDNA Synthesis SuperMix (TransGen Biotech, Beijing, China).

RT-qPCR reaction analysis was carried out in a total 20 μL volume containing 7.8 μL of RNase-free water, 10 μL of 2 × SYBR Premix (Tli RNaseH Plus; Takara Bio), 0.6 μL of 10 μM of each forward and reverse gene-specific primer, and 1.5 μL of fold diluted cDNA template. RT-qPCR parameters were followed by The *NbEF1-α* of the housekeeping gene used for normalization. The condition follows an initial denaturation step at 95°C for 3 min, 40 cycles of 95°C denaturation for 15 s, 60°C annealing for 20 s, and 72°C extension for 20 s. The expression of the target genes in different samples was calculated using the formula 2^–ΔΔCT^ mothed and presented as a value relative to that of the control treatment. The RT-qPCR experiment was repeated twice, and each contained three technical replicates. All of the primers are listed in Supplementary Table S1.

### 2.8. Growth and development of *Botrytis cinerea* by KRS005

Confocal microscopy observations of *B. cinerea* treated by KRS005 fermentation broth were used to reveal the effects on mycelial morphology. The strain KRS005 was cultured in LB-OP medium at 28°C for 3 days with 200 rpm, cell-free supernatant was collected from culture filtrate by centrifuging at 8,000 g for 10 min. Cell-free supernatant was mixed with PDA medium for incubation, and a hypha patch of *B. cinerea* was placed in the center of the plate after the medium had solidified the culture plate and incubated at 25°C for 24 and 48 h to measure the mycelial diameter. The change in membrane permeability was used to determine the transmembrane conductivity. Mycelial of *B. cinerea* from a six-day-old colony was suspended in the solution containing 0.01% (v/v) Tween—80. Next, cell-free supernatant of KRS005 was added to *B. cinerea* mycelial suspension at concentrations of 5%, 10%, 15%, and 100% (v/v), respectively. The electric conductivity of mycelial suspensions of *B. cinerea* was measured after 40, 100, 200, 300, 500, 1,440, and 2,880 min. The effect of cell membrane permeability of *B. cinerea* by measuring electrolyte leakage, using the modified methods (Zhang et al., 2016; Liu et al., 2017), followed by measurement of solution ionic conductivity using a conductivity meter (Mettler-Toledo, Shanghai, China) with a Probe LE703. Each treatment had at least three biological repeats, and the assay was replicated three times.

## 3. Results

### 3.1. Molecular-based to identify the taxonomy of an endophyte microbe from cotton

**Figure 1:**
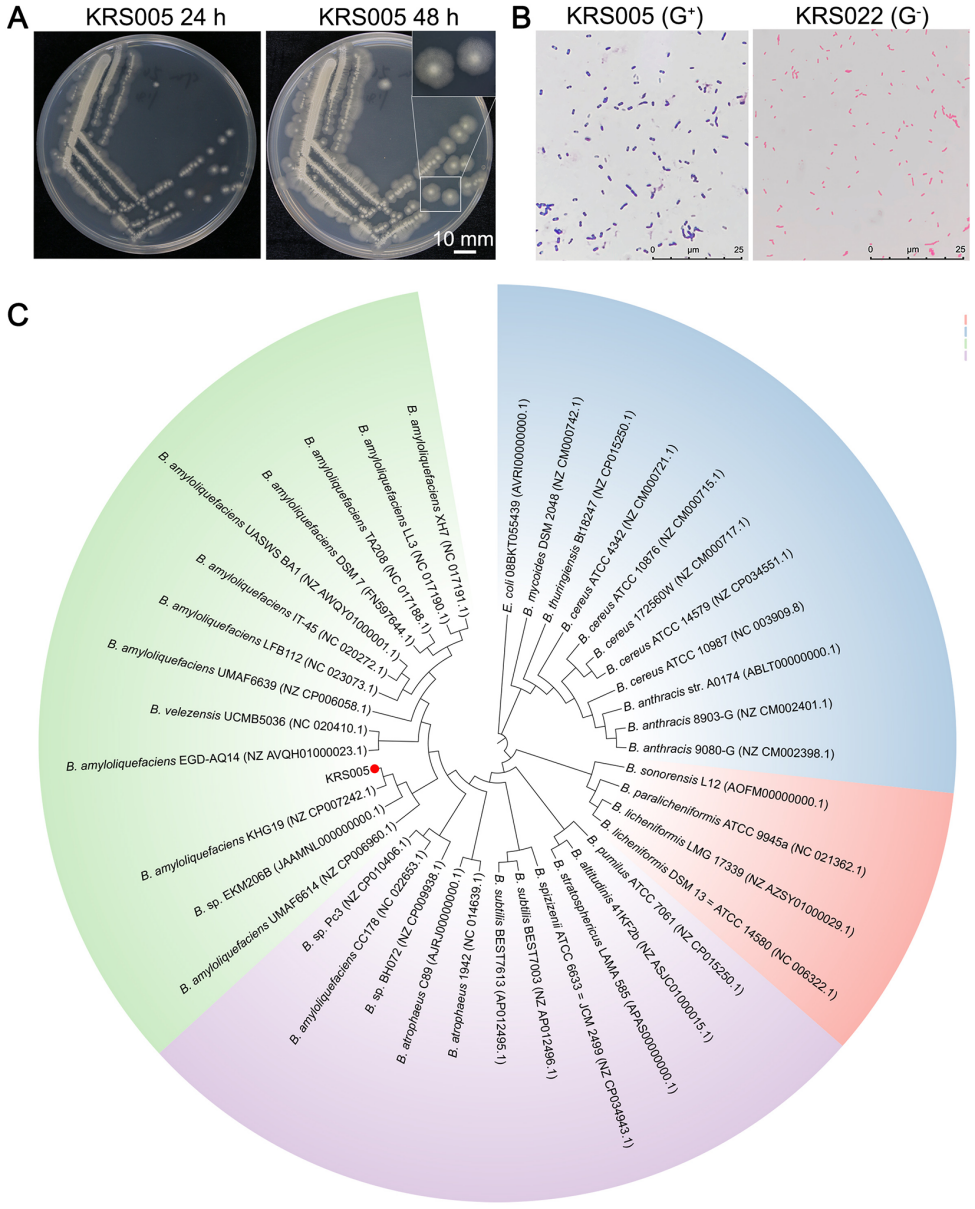
Identify the taxonomy of an endophyte microbe KRS005 from cotton stem. **(A)** Morphological characteristics of strain KRS005 on LB medium plate cultured 12 and 24 h. **(B)** Gram staining observation under an optical microscope, *Pseudomonas alcaligenes* was severed as gram-negative control. **(C)** Phylogenetic tree of the KRS005 strain and its homolog bacterial species by searching with the *gyrB*, *gyrA,* and *rpoB* nucleotide sequences. The phylogenetic tree was performed by MLSA-MLST methods using the connection of *gyrB*, *gyrA,* and *rpoB* sequence, with MEGA6.0 software. *Escherichia coli* (08BKT055439) was used as an outgroup to root the tree. The bootstrap analysis was performed with 10,000 replications.

In this study, we screened the potential biocontrol microorganisms isolated from the cotton stem, named KRS005. The colony of strain KRS005 is pale yellow in color, suborbicular, and with a dry surface, which also exhibited an irregular edge at 12 and 24 h after culturing on Luria-Bertani (LB) medium at 28°C (Figure 1A). Isolate KRS005 was identified as a gram-positive strain by gram staining (Figure 1B). To further identify the taxonomy of KRS005, the phylogenetic analysis was performed by multilocus sequence analysis and typing (MLSA–MLST). In detail, three loci of type II topoisomerase DNA gyrase alpha-submit gene (*gyrA*), type II topoisomerase DNA gyrase beta-subunit gene (*gyrB*), and RNA polymerase beta-subunit gene (*rpoB*) were amplified from the KRS005 genomic DNA, respectively, using the specific primers listed in Supplementary Table S1. The sequence analysis used the BLASTn from National Center for Biotechnology Information (NCBI). The phylogenetic analysis involved in the MLSA-MLST (tandem loci as *gyrB* − *gyrA* − *rpoB*) was performed with a high bootstrap value of 99% (Figure 1C). Together, these results suggested that the KRS005 isolated from cotton stem is a gram-positive strain and belongs to *Bacillus amyloliquefaciens.*

### 3.2. Physiological and biochemical properties of KRS005

**Figure 2:**
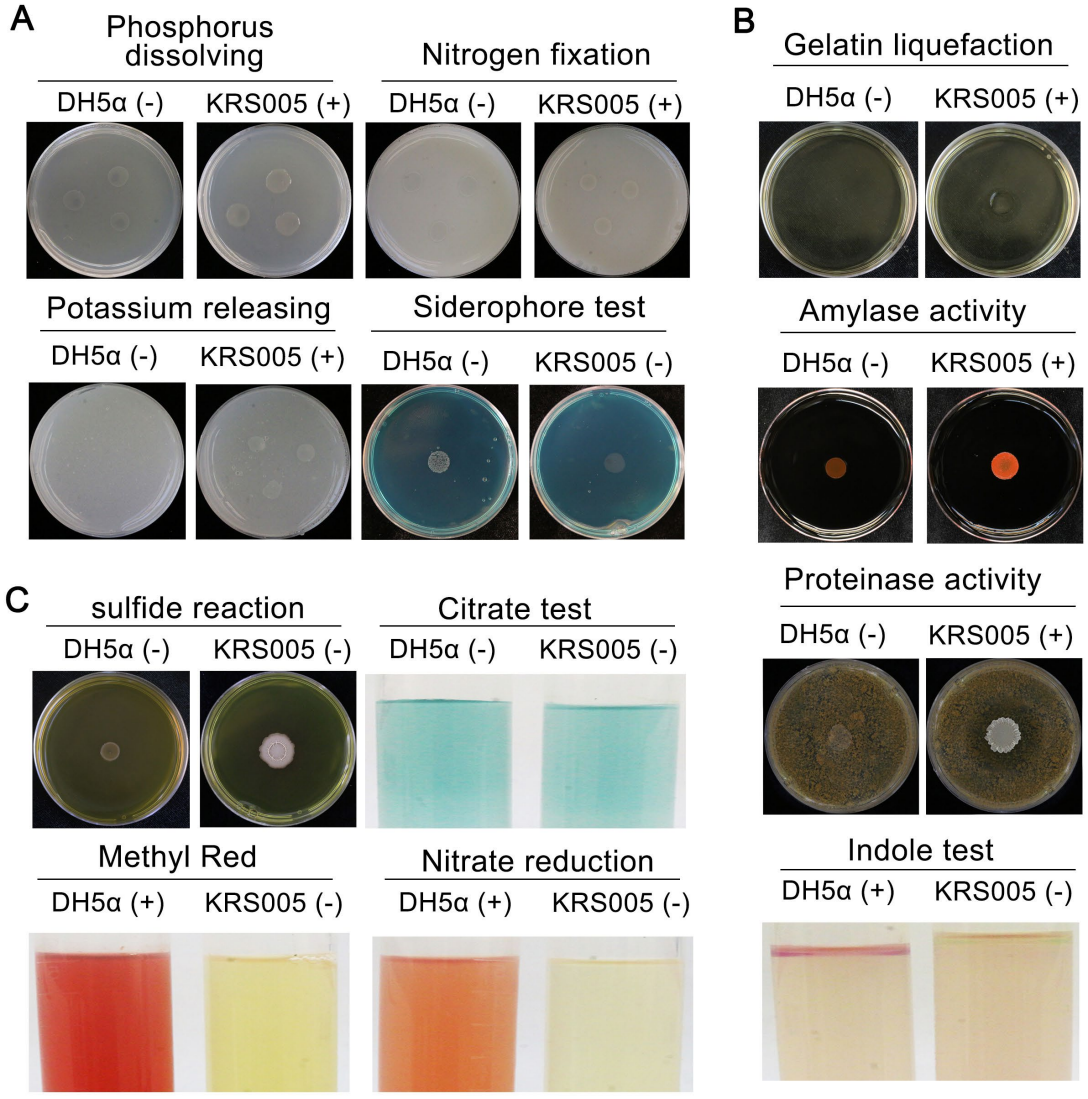
Physiological and biochemical characteristics of strain KRS005. **(A)** Nitrogen fixation, phosphate and potassium solubilization ability, and siderophore production of KRS005 were determined. **(B)** Gelatin liquefaction, amylase activity, proteinase activity, and indole assays. **(C)** Sulfide reaction, citrate, methyl red, and nitrate reduction were determined. *E. coli* DH5α was severed as control. “+” indicates that the result is positive reaction, “−” indicates that the result is negative reaction. In all of the physiological and biochemical tests, *Escherichia coli* DH5α was used as a control, each treatment was conducted in three replicates, and every test was performed at least three times.

To further identify KRS005 as *B. amyloliquefaciens*, the analysis of physiological and biochemical characteristics involved in siderophore production, phenylalanine deamination, indole production, methyl red (MR) reaction, nitrate reduction, gelatin liquefaction, and hydrogen sulfide were performed. The results indicated KRS005 has the ability of phosphate utilization, hydrogen sulfide production, nitrate reduction, and gelatin liquefaction (Figure 2A). The clearing ring of agar plates containing starch around the colony exhibits its ability of starch hydrolysis. Compared to the negative control, DH5α, the physiological tests showed that the strain KRS005 could hydrolyze starch and protein, suggesting the strain can produce amylase and protease. In addition, the indole enzyme was positive (Figure 2B). In comparison, the screening tests for the formation of sulfide tests, methyl red reaction, and nitrate reduction were negative. KRS005 can utilize citric acid as a carbon source (Figure 2C). According to the physiological and biochemical properties of the strain, the property of KRS005 is consistent with those strains of *B. amyloliquefaciens* in previous reports.

### 3.3. Broad-spectrum inhibitory activity of KRS005 on plant pathogenic fungi

**Figure 3:**
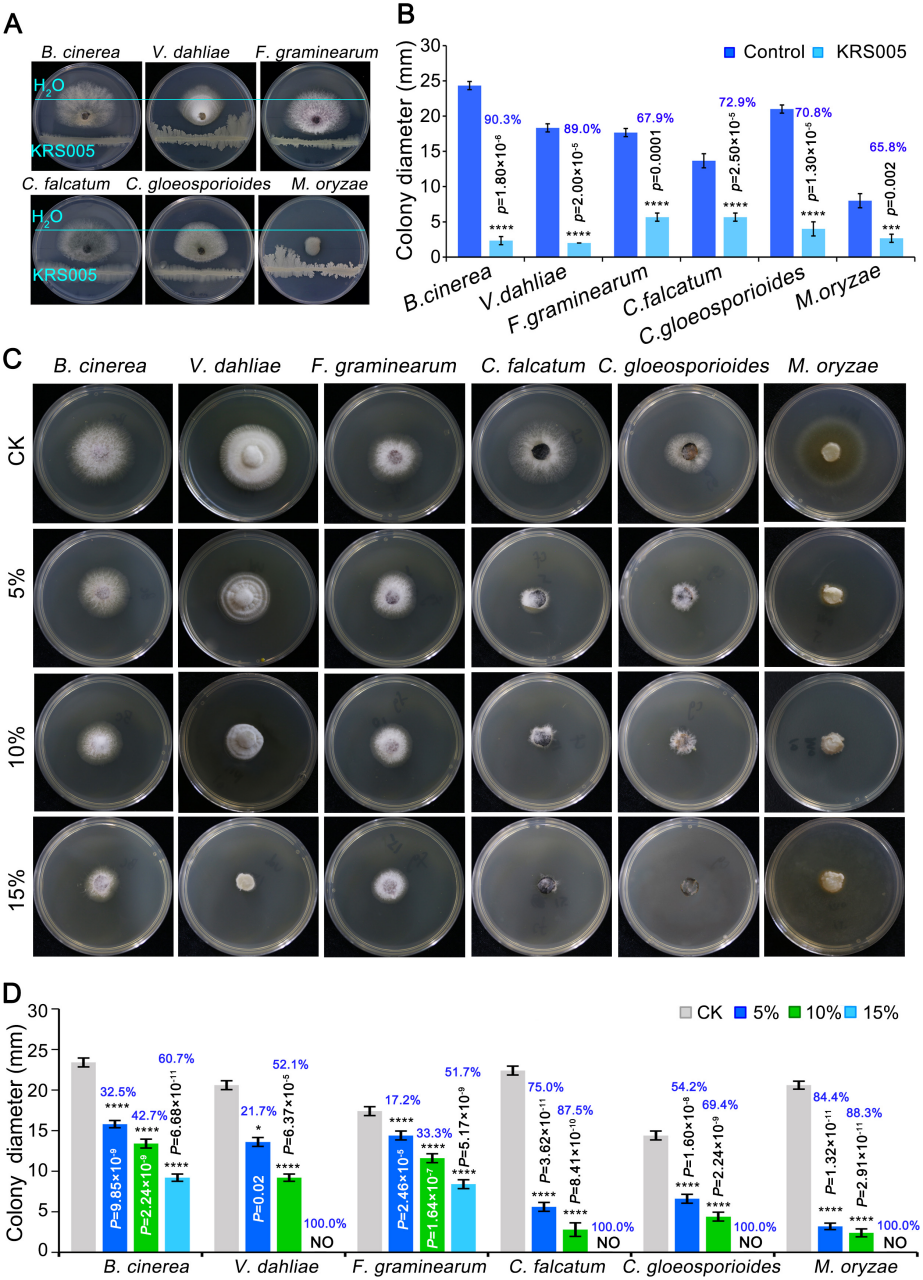
The broad-spectrum activity of KRS005 against plant pathogenic fungi. **(A)** *In vitro* effect of KRS005 on the plant pathogens. *Botrytis cinerea* and *Verticillium dahliae, Fusarium graminearum*, *Colletotrichum falcatum*, *Colletotrichum gloeosporioides*, and *Magnaporthe oryzae* were cultured on potato dextrose agar (PDA) medium in the presence of KRS005. **(B)** The inhibition rate of KRS005 against plant pathogenic fungi. **(C)** Phenotypes for KRS005 cell-free supernatant against plant pathogenic fungi. **(D)** The inhibition rate of KRS005 cell-free supernatant against plant pathogenic fungi. Blue text indicates the inhibition rate. Error bars represent standard deviations. *, ***, and **** indicate the significant difference at *p* < 0.05, *p* < 0.001, and *p* < 0.0001, respectively, according to the unpaired Student’s *t*-test.

To assess whether the strain KRS005 had a broad-spectrum antifungal activity, six pathogenic fungi were detected by dual confrontation culture. The results showed that strain KRS005 had different degree of inhibition on the mycelial growth of six pathogenic fungi, of which the inhibition rate of *B. cinerea* was the highest, as high as 90.3%, and *Verticillium dahliae*, *Colletotrichum falcatum*, and *C. gloeosporioides* were 89%, 72.9%, 70.8%, respectively, compared to control (Figures 3A,B). For investigating antifungal efficiency, the cell-free supernatant of KRS005 was added to the PDA plates with 5, 10, and 15% (v/v), respectively. PDA plate without the cell-free supernatant was used as control. The colony diameters of different pathogenic fungi were measured to assess the antifungal activity. The results demonstrated that different concentrations (5%, 10%, and 15% (v/v), respectively) of cell-free supernatant of KRS005 had varying degrees of inhibitory effects on the pathogen fungi tested, and the inhibitory effect on pathogenic fungi was enhanced with increased concentration. Among which the 15% cell-free supernatant of KRS005 could completely inhibit the growth of *V. dahliae*, *C. gloeosporioides*, *C. falcatum*, and *M. oryzae*, respectively, and the inhibition rate on *B. cinerea* was up to 60.7% (Figures 3C,D). In addition, the inhibition rate of *B. cinerea* was positively correlated with the proportion of cell-free supernatant. Taken together, these results indicated that strain KRS005 had a broad-spectrum inhibitory effect on various phytopathogenic fungi, especially in *B. cinerea*. Moreover, its cell-free supernatant also demonstrated a significant inhibitory effect, indicating that KRS005 is a potential biocontrol agent.

### 3.4. The biocontrol effect of strain KRS005 on gray mold

**Figure 4:**
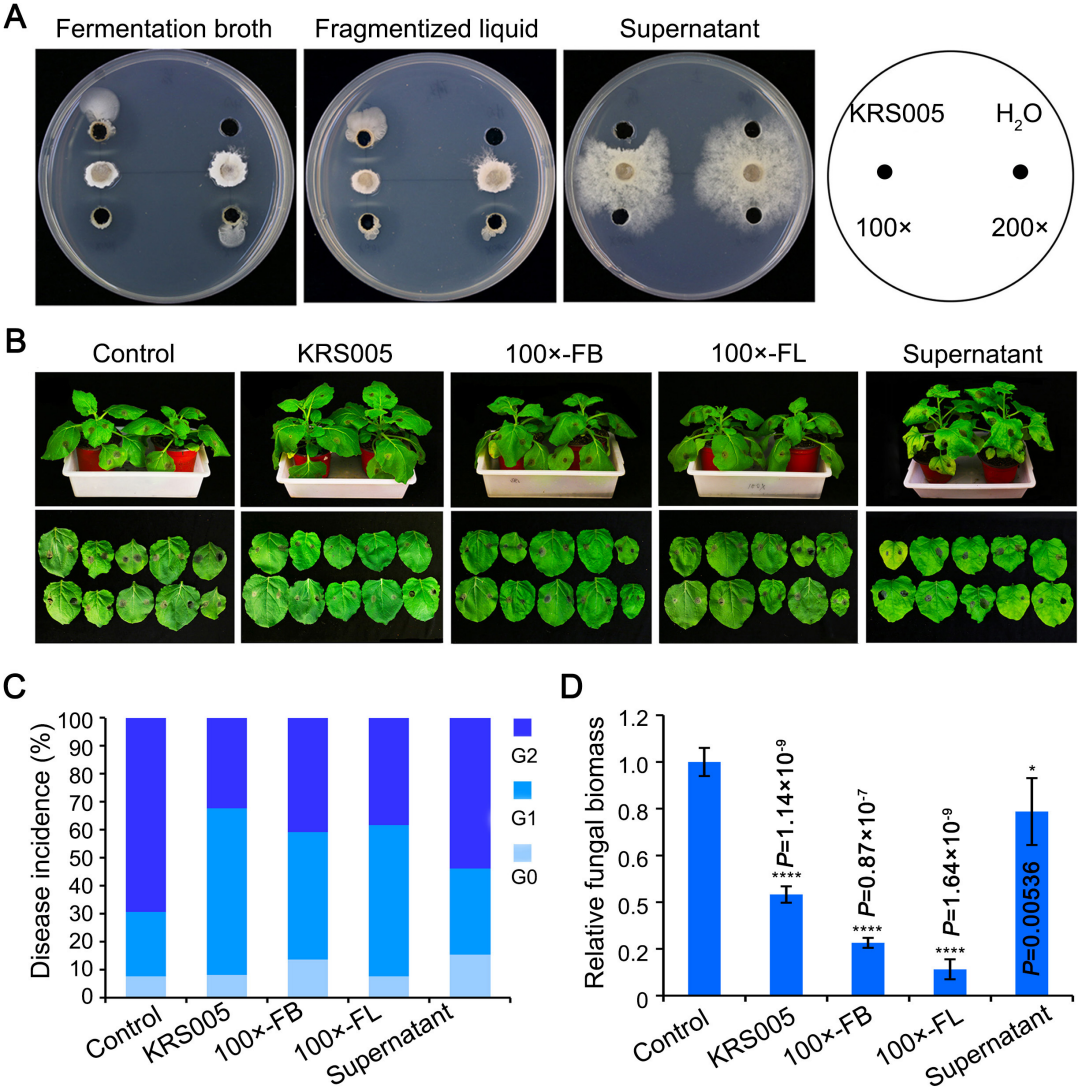
Effect of different treatments of KRS005 on gray mold. **(A)** Antifungal effect of different KRS005 culture filtrate on *Botrytis cinerea* growth. **(B)** Different KRS005 culture filtrate inhibits gray mold lesions on tobacco leaves at 4 days post-inoculation (dpi). LB: LB broth, 100 × FB: diluted 100-fold of fermentation broth, 100 × FL: diluted 100-fold of fragmentized liquid, Supernatant: cell-free supernatant. **(C)** Disease incidences of infected leaves at 4 dpi. The diameter of the lesion was decreased in KRS005-treated leaves compared with the control. The grade of disease was classified as 0, 1, 2, with the lesion diameter as 0 mm, 1~10 mm, and more than 10 mm, respectively. **(D)** The fungal biomass in tobacco leaves by quantitative PCR (qPCR) at 4 dpi. Error bars represent standard deviations, and asterisks *, **, ***, and **** indicate the significant difference at *p* < 0.05, *p* < 0.01, *p* < 0.001, and *p* < 0.0001, respectively, according to unpaired Student’s *t*-test.

As described above, the screening for antagonistic activity revealed that KRS005 reduced the mycelial growth of *B. cinerea* by 90.3% (Figure 3B). Thus, the inhibitory activity of isolated KRS005 to plant pathogenic fungi, especially *B. cinerea,* was further studied *in vivo*. First, the fermentation broth (FB), fragmentized liquid (FL), and cell-free supernatant of KRS005 were added to the PDA plates at different dilution ratios, respectively, and the antifungal activity was observed in all treatments, which indicated that these liquids had good antifungal activity. Especially, FB and FL still keep a stable activity at the dilution of 100 and 200 folds, respectively (Figure 4A). Moreover, the isolate KRS005 FB was sprayed on the four-week-old tobacco leaves for 24 h, and inoculated *B. cinerea* on leaves for the lesion diameter measurement of 3 days. The results showed that there was a significant reduction in the size of disease development of *B. cinerea* in KRS005-treated tobacco leaves, including KRS005 FB, FL, and cell-free supernatant, compared with control (Figure 4B). According to the diameter of the leaf lesion, the disease was evaluated on a 0–2 scale, of which Grade 0 indicates 0 mm of leaf lesion diameter, Grade 1 indicates 0–10 mm of leaf lesion diameter, and Grade 2 was equal or greater than 10 mm. These results demonstrated that the lesion diameter of gray mold on KRS005-treated (FB, FL, cell-free supernatant) tobacco leaves was significantly lower than that of the control (Figure 4C). Fungal biomass was analyzed by quantitative PCR (qPCR), and the results showed that the reproduction of fungi decreased significantly under 100-fold dilution of KRS005 FB treatment (Figure 4D). The lesion diameter was decreased when a high proportion of KRS005 FB was used, and then the KRS005 had a strong biocontrol effect on gray mold after dilution (Figures 4B–D). Together, these results indicated that *B. amyloliquefaciens* KRS005 has a prospect in the prevention and control of tobacco gray mold.

### 3.5. The KRS005 triggers plant immunity response

**Figure 5:**
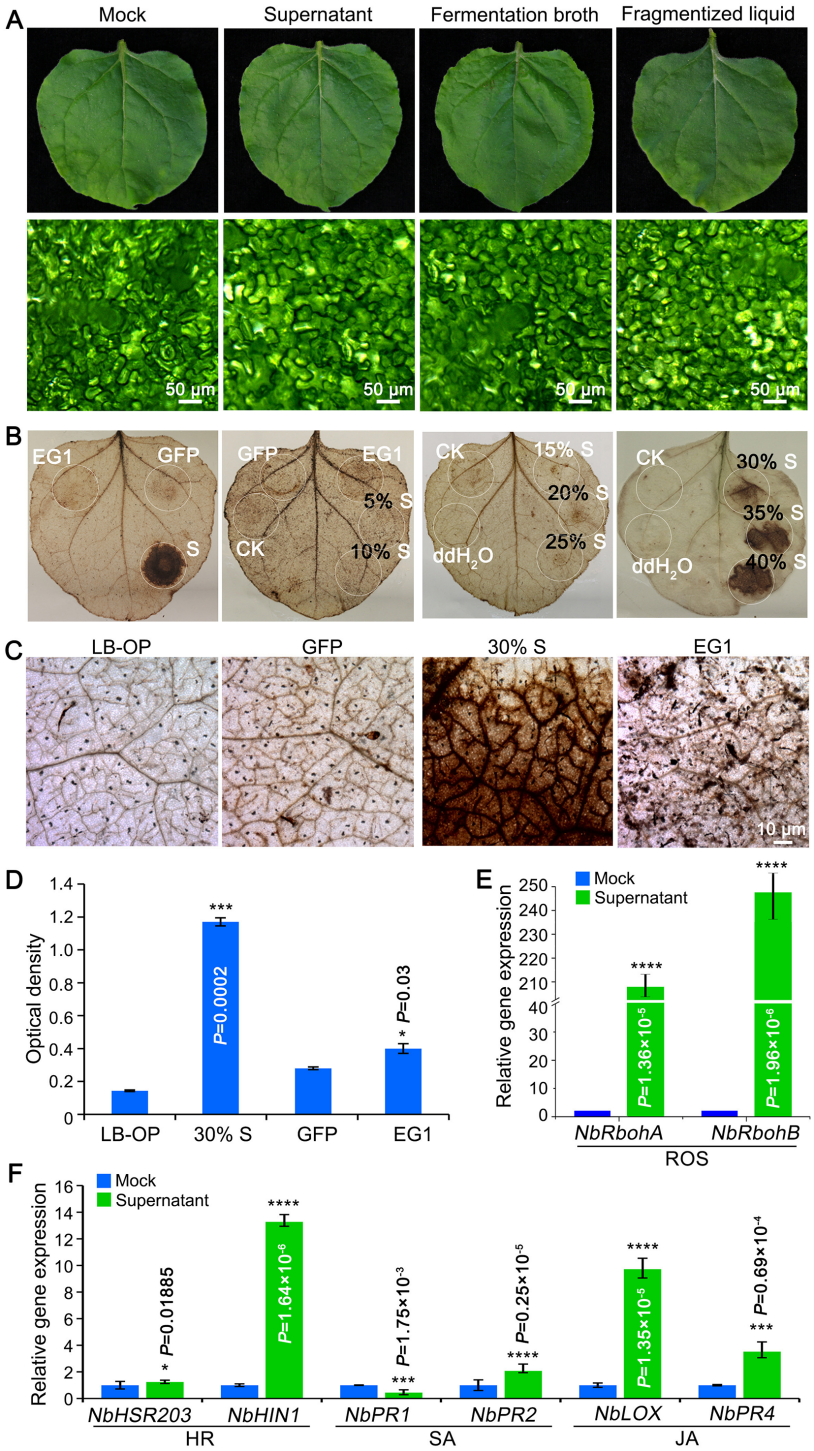
KRS005 triggers plant immunity response. **(A)** Phytotoxicity of KRS005 culture filtrate on tobacco leaves after 12 h (Scale bars = 50 μm). **(B,C)** DAB staining observations of tobacco leaves treated with KRS005 cell-free supernatant. LB-OP, Luria-Bertani optimized medium optical density; GFP, Green fluorescent protein used for negative control; EG1, effector VdEG1 that triggers cell death (Scale bars = 10 μm). **(D)** DAB optical density (OD) data. **(E,F)** Expression of defense-related genes in tobacco leaves of uninoculated or uninoculated cell-free supernatant-treated or mock-treated tobacco plants 6 h after treatments. Error bars represent standard deviations, and asterisks *, ***, and **** indicate the significant difference at *p* < 0.05, *p* < 0.001, and *p* < 0.0001, respectively, according to unpaired Student’s *t*-test.

The safety of biocontrol strain is an essential precondition for further application. The pathogenicity of KRS005 was examined by spraying FB on tobacco leaves, and mesophyll cells were observed using the stereomicroscope. The results demonstrated no virulence on leaves, and the mesophyll cells were intact (Figure 5A). The production of reactive oxygen species (ROS) is a common immune response in plants. To explore whether tobacco resistance was triggered, the concentration of ROS was determined by DAB staining. The cell-free supernatant of KRS005 was injected into the tobacco leaves, and the typical PAMPs VdEG1 and GFP were severed as positive and negative controls, respectively. These assays indicated that KRS005 cell-free supernatant could induce a strong ROS response at 30, 35, 40, and 100% (v/v) concentrations, respectively (Figure 5B). The phenotype observation and optical density of the leaves’ injection area also showed that the 30% (v/v) KRS005 cell-free supernatant could induce a higher ROS response than the positive control VdEG1 (Figures 5C,D). Moreover, the expression level of ROS response-related marker genes was detected by RT-qPCR. The *NbRbohA* and *NbRbohB* were upregulated (Figure 5E).

In addition, the expression profiles of SA (*NbPR1*, *NbPR2*), JA (*NbLOX*, *NbPR4*), and HR-related genes (*NbHSR203*, *NbHIN1*) were detected by RT-qPCR after spraying the KRS005 cell-free supernatant in tobacco plants. According to the results, cell-free supernatants elicited defense responses in tobacco plants, as the expression of defense-related genes was significantly upregulated in plants treated with cell-free supernatants compared to plants inoculated with water. KRS005 could trigger plant immunity by ROS response and HR, SA/JA-related signal pathway.

### 3.6. The cell-free supernatant of KRS005 influences the growth and morphology of *Botrytis cinerea*

**Figure 6:**
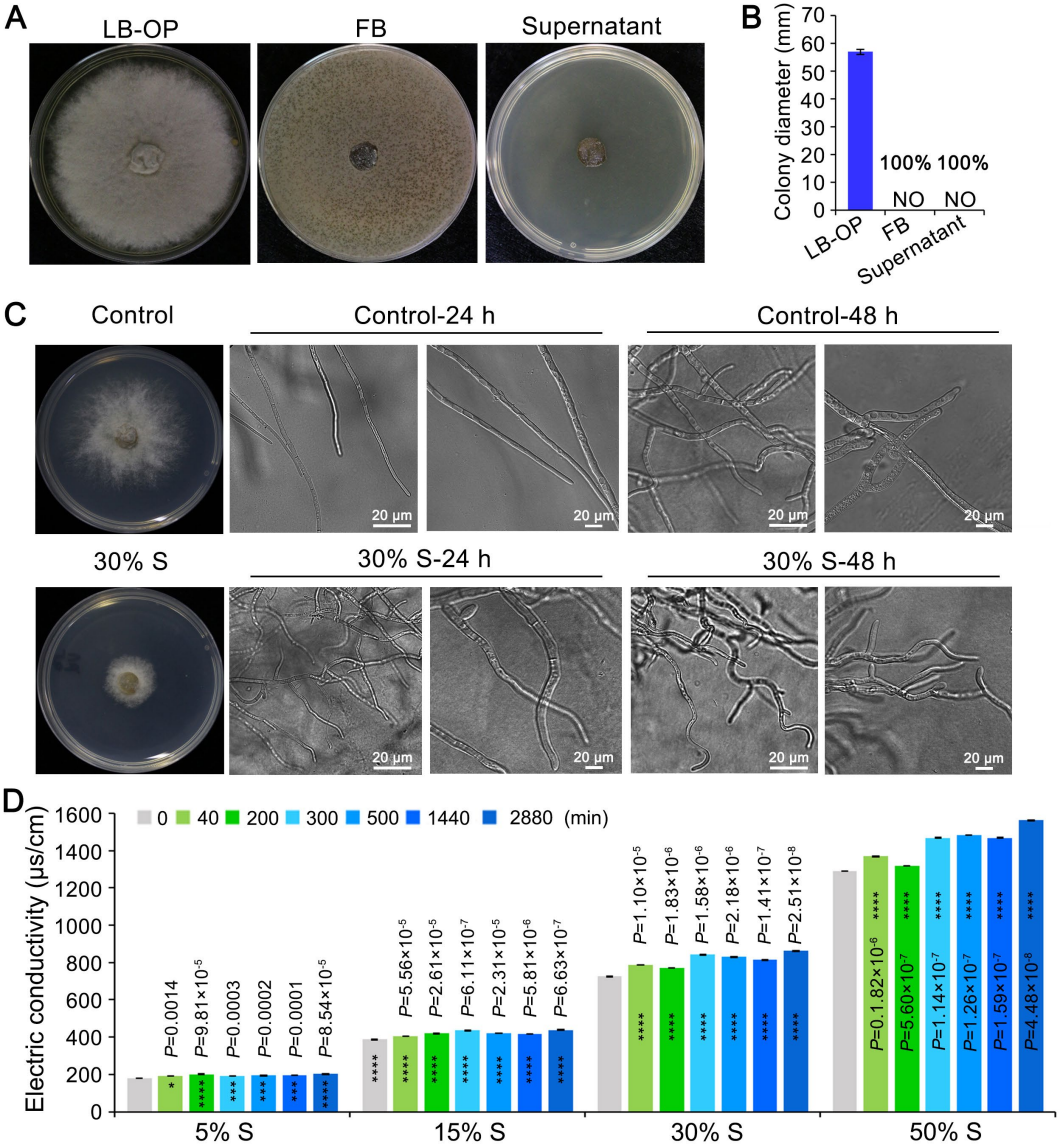
The KRS005 influences the growth and morphology of *Botrytis cinerea*. **(A)** The cell-free supernatant of KRS005 significantly inhibited the mycelial growth of *B. cinerea*. LB-OP: PDA medium was mixed with optimized Luria-Bertani at 2:1, FB: PDA medium was mixed with fermentation broth at 2:1, Supernatant: PDA medium was mixed with fermentation broth at 2:1. **(B)** *Botrytis cinerea* colony diameter from three repeated experiments. **(C)** Inhibition of the plate fungistatic experiment of the normal mycelium, and mycelium treated with 30% of cell-free supernatant. LB medium as negative control. S: cell-free supernatant. The fungal structures were observed with 10 × and 40 × objectives, respectively (Scale bars = 20 μm). **(D)** Effect of KRS005 cell-free supernatant on cell membrane permeability of *B. cinerea.* Error bars represent standard deviations. The asterisks *, ***, and **** indicate the significant difference at *p* < 0.05, *p* < 0.001, and *p* < 0.0001, respectively, according to the unpaired Student’s *t*-test.

To examine the antifungal activity of the strain KRS005 against the mycelial growth of *B. cinerea*, the KRS005 FB or cell-free supernatant was added to the LB plate. Then the *B. cinerea* was inoculated on the center of the PDA plate. Both FB and cell-free supernatant completely inhibit *B. cinerea* growth however, the growth of *B. cinerea* on PDA plate with LB was not affected (Figures 6A,B). The morphological development of *B. cinerea* mycelia was observed under cell-free supernatant stress. The growth of *B. cinerea* on PDA plate containing the same amount of LB was severed as control. *Botrytis cinerea* mycelia were significantly (*p* < 0.0001) inhibited after 24 h of 30% (v/v) cell-free supernatant stress culture. The cell-free supernatant of KRS005 caused tube length reduction and led to abnormal development (Figure 6C). With the increase in incubation time (48 h), the distortion of *B. cinerea* mycelia became more obvious. The mycelia treated with the 30% (v/v) cell-free supernatant were irregularly reticulated and uneven in thickness, and the top of them was crooked, twisted, and shriveled. On the contrary, the *B. cinerea* mycelia of the control group were uniform in thickness and slender, with fewer branches and a good growth state (Figure 6C).

In this study, KRS005 could damage the cell membrane of *B. cinerea* was detected by electric conductivity determination. An increase in electrical conductivity was observed over time when *B. cinerea* was exposed to increasing concentrations of cell-free supernatant at 5%, 15%, 30%, and 50% (v/v), respectively, which suggested the cell membrane permeability of *B. cinerea* was increased rapidly in response to increasing concentrations of cell-free supernatant (Figure 6D). Together, these assays suggested that KRS005 could directly inhibit the morphological development of *B. cinerea* mycelia, which may result in cell membrane damage and increased permeability of *B. cinerea*.

## 4. Discussion

Fungicides are currently the main strategy to control gray mold caused by the plant fungal pathogen *B. cinerea*, and account for 10% of the market share (Dean et al., 2012). *Botrytis cinerea* is one of the major fungal pathogens, causing harvest losses in a wide range of tobacco (Dean et al., 2012; Chowdhury et al., 2015). However, for the sake of environmental pollution and chemical pesticide resistance, the demand for biological control agents is increasing, which are usually used to reduce the infestation of insect pests and plant pathogens. In recent years, *Bacillus* sp. has been considered one of the best biogenic agents for controlling plant pathogens, which are mainly applied as solid or liquid fertilizers (Javed et al., 2020) and have exhibited excellent control effects on diseases in cucumber (e.g., gray mold and powdery mildew), strawberry (e.g., gray mold and downy mildew), grapes (e.g., gray mold and white rot), pepper (e.g., gray mold and leaf spot), and other crops (Kim et al., 2013; Hamaoka et al., 2021; Kazerooni et al., 2021; Nifakos et al., 2021). The biological control of the gray mold satisfies environmental protection because it leaves low residue on plants. It is worth noting that the reasons that the bacterial strains from the genus *Bacillus* are characterized as biocontrol agents are based on their ability not only to control pathogen propagation but also to improve plant growth and health (Chowdhury et al., 2015). Of which, *B. amyloliquefaciens* as a biological fertilizer in agriculture, and plant growth promotion (PGP) mechanism has been extensively investigated. Some strains were reported to have the functions of siderophore production, phosphate/potassium solubilization, and nitrogen fixation, and change the soil microbial community that improves the effectiveness of mineral nutrients and the root environment for crop growth. Moreover, there were other strains that could produce hormones and volatile organic compounds (VOCs) related to plant cell and root growth, promoting plant nutrient acquisition. For instance, *B. amyloliquefaciens* DHA55 exhibited significant antifungal activities against *Fusarium* on the root surface of watermelon (Al-Mutar et al., 2023). Strain Oj-2.16 exhibited a high inhibition rate against *Verticillium dahliae* in tomato of up to 89.26% and promoted the growth of tomato seedlings (Pei et al., 2022). Similarly, *Bacillus* has a control effect against gray mold.

In this study, an antagonistic strain KRS005 was isolated from cotton tissue, and its potential as a biological control agent was investigated. In order to identify the strain, morphological, physiological, and biochemical characteristics were studied by standard methods (Moyes et al., 2009). Strain KRS005 was identified as *B. amyloliquefaciens* by gram staining and phylogenetic analysis (Figure 1), and the biocontrol efficacy of *B. amyloliquefaciens* against a variety of plant diseases was studied (Zheng et al., 2018). It has been reported in previous studies that *Bacillus* has a role in the control of fungal diseases. The control mechanisms mainly include inhibiting fungal growth and improving plant disease resistance. Enzyme activity showed that secondary metabolites hydrolyze the cell wall of pathogenic fungi (Aktuganov et al., 2007; Chowdhury et al., 2015). Gelatinase, protease, and amylase were detected in physiological and biochemical experiments, which may help isolate KRS005 to catalyze and hydrolyze the cell wall of pathogenic fungi (Figure 2). The starch hydrolysis assay measured the ability of bacterial isolates to produce α-amylase, an enzyme that hydrolyzes polysaccharides such as starch (Simair et al., 2017). Inhibition of fungal growth by biological control agents was assessed by measuring the relative radius of mycelium growth (Khan et al., 2018; Besset-Manzoni et al., 2019; Dai et al., 2021). In our study, the bacteriostatic test was carried out by plain scratching or coating method. *In vitro*, *B. amyloliquefaciens* showed high activity as a biocontrol agent against gray mold (Figure 3). In addition, KRS005 fermentation broth diluted 100 times can effectively reduce the occurrence of tobacco gray mold (Figure 4). Compared with the fermentation broth of KRS005, the cell-free supernatant of KRS005 has shown a lower inhibitory rate on the mycelial growth of *B. cinerea*. This implied that KRS005 may inhibit pathogen fungi by volatile organic compounds (VOCs), which will provide a reference for future work.

The KRS005 fermentation broth caused the mycelium of *B. cinerea* to form a balloon-like structure, resulting in the inhibition of mycelium growth. In addition, the KRS005 fermentation broth inhibited and delayed the elongation of the germ tube. The possible role strain mode includes fungal cell wall degradation. The electrolyte leakage test confirmed that the activities of KRS005 cell wall degradation enzymes had a degrading effect on the cell wall of *B. cinerea* (Figure 6). Moreover, the cell-free supernatant of KRS005 showed inhibitory effects on different fungal species such as *B. cinerea* and *V. dahliae* (Figure 3). However, the active antifungal metabolites were not analyzed (Ju et al., 2014).

On the other hand, we also investigated whether strain KRS005 inhibits the growth of pathogenic fungi by triggering plant immunity. Beneficial strain-induced defense responses controlled by signal networks, such as reactive oxygen species (ROS) bursts, HR, SA, and JA, play an important role (Hammond-Kosack and Parker, 2003). In addition, a large amount of evidence has shown that plant-microbial synergy has a positive effect on improving plant disease resistance and stress resistance, and promoting plant growth and yield. For instance, *B. amyloliquefaciens* strain NC6 could induce typical HR, activate ROS bursts in tobacco leaves, and enhance tobacco resistance to *B. cinerea* by triggering the upregulation of defense-related genes and accumulation of antimicrobial compounds (Wang et al., 2016). Further indicate that KRS005 may also trigger the expression of some plant defense genes responsive to different phytohormonal pathways (Basit et al., 2021). In tobacco leaves treated with cell-free supernatant, the ROS/HR-responsive genes, *NbHIN1*, *NbRbohA*, and *NbRbohB,* were strongly upregulated, while the expression of SA/JA-responsive genes, *NbPR2*, *NbLOX*, and *NbPR4*, were only slightly induced (Figure 5). The reduced pathogen population might be due to the defense responses evoked by strain KRS005 because the induction of defense-related gene expression accompanied the suppression of pathogen growth in the tissue of KRS005-treated plants. After further study, KRS005 enhances tobacco disease resistance to *B. cinerea* by triggering resistance-related gene up-regulation. These results indicate that our research helps to elucidate the mechanisms of KRS005-induced systemic resistance in tobacco. Hence, the effect of strain KRS005 on tobacco defense response showed that it meets the production safety standards.

In conclusion, although the substances released by the strain KRS005 have not been identified, it has been demonstrated that the most polar substances in this extract had antifungal action. The results of this integrated approach conclude that the strain KRS005 could be used as a biocontrol agent through its ability to inhibit the growth of fungal pathogens and reduce the severity of fungal infections *in vitro*. *Bacillus amyloliquefaciens* KRS005 is likely a good candidate microbial resource to be tested under greenhouse conditions to verify the biocontrol effect and other economically important crops.

## Data availability statement

The original contributions presented in the study are included in the article/Supplementary material, further inquiries can be directed to the corresponding authors.

## Author contributions

J-YC, X-FD, X-JZ, and DW conceived and designed the experiments. H-YQ performed the experiments. H-YQ and DW analyzed the data and wrote the initial draft. JS performed the strain isolation and screening. DH, MA, and J-YC reviewed and edited the manuscript. All authors contributed to the article and approved the submitted version.

## Funding

This work was supported by the National Key Research and Development Program of China (2022YFD1400300) and 2019 Youth Cultivation Projects in the Basic Research Business Funds of Provincial Universities (1354MSYQN025).

## Conflict of interest

The authors declare that the research was conducted in the absence of any commercial or financial relationships that could be construed as a potential conflict of interest.

## Publisher’s note

All claims expressed in this article are solely those of the authors and do not necessarily represent those of their affiliated organizations, or those of the publisher, the editors and the reviewers. Any product that may be evaluated in this article, or claim that may be made by its manufacturer, is not guaranteed or endorsed by the publisher.

## Footnotes

^1^ https://blast.ncbi.nlm.nih.gov/Blast.cgi

## References

[ref1] Agarbati A. Canonico L. Pecci T. Romanazzi G. Ciani M. Comitini F. (2022). Biocontrol of non-saccharomyces yeasts in vineyard against the gray mold disease agent *Botrytis cinerea*. Microorganisms. 10:200. doi: 10.3390/microorganisms10020200, PMID: 35208653 PMC8874649

[ref2] Aktuganov G. E. Galimzianova N. F. Melent'ev A. I. Kuz'mina L. (2007). Extracellular hydrolases of strain *Bacillus* sp. 739 and their involvement in the lysis of micromycete cell walls. Mikrobiologiia 76, 471–479. doi: 10.1134/S0026261707040054, PMID: 17974203

[ref3] Al-Mutar D. M. K. Alzawar N. S. A. Noman M. Azizullah M. Li D. Song F. (2023). Suppression of fusarium wilt in watermelon by *Bacillus amyloliquefaciens* DHA55 through extracellular production of antifungal Lipopeptides. J Fungi 9:336. doi: 10.3390/jof9030336, PMID: 36983504 PMC10053319

[ref4] Apine O. A. Jadhav J. P. (2011). Optimization of medium for indole-3-acetic acid production using *Pantoea agglomerans* strain PVM. J. Appl. Microbiol. 110, 1235–1244. doi: 10.1111/j.1365-2672.2011.04976.x, PMID: 21332896

[ref5] Basit A. Farhan M. Abbas M. Wang Y. Zhao D. G. Mridha A. U.. (2021). Do microbial protein elicitors PeaT1 obtained from *Alternaria tenuissima*and PeBL1 from *Brevibacillus laterosporus*enhance defense response against tomato aphid (*Myzus persicae*). Saudi J Biol Sci. 28, 3242–3248. doi: 10.1016/j.sjbs.2021.02.063, PMID: 34121861 PMC8176006

[ref6] Bernardes M. F. F. Pazin M. Pereira L. C. Dorta D. J. (2015). “Toxicology studies-cells, drugs and environment,” in Impact of Pesticides on Environmental and Human Health. Ed. A. C. Andreazza (London, United Kingdom: Intech Open Limited 5 Princes Gate Court), 195–233.

[ref7] Besset-Manzoni Y. Joly P. Brutel A. Gerin F. Soudière O. Langin T.. (2019). Does in vitro selection of biocontrol agents guarantee success in planta? A study case of wheat protection against *fusarium* seedling blight by soil bacteria. PLoS One 14:e0225655. doi: 10.1371/journal.pone.0225655, PMID: 31805068 PMC6894788

[ref8] Bindschedler L. V. Dewdney J. Blee K. A. Stone J. M. Asai T. Plotnikov J.. (2006). Peroxidase dependent apoplastic oxidative burst in *Arabidopsis* required for pathogen resistance. Plant J. 47, 851–863. doi: 10.1111/j.1365-313X.2006.02837.x, PMID: 16889645 PMC3233234

[ref9] Bonaterra A. Badosa E. Daranas N. Francés J. Roselló G. Montesinos E. (2022). Bacteria as biological control agents of plant diseases. Microorganisms. 10:1759. doi: 10.3390/microorganisms10091759, PMID: 36144361 PMC9502092

[ref10] Chen X. Wang Y. Gao Y. Gao T. Zhang D. (2019). Inhibitory abilities of *Bacillus* isolates and their culture filtrates against the gray mold caused by *Botrytis cinerea* on postharvest fruit. Plant Pathol. J. 35, 425–436. doi: 10.5423/PPJ.OA.03.2019.0064 31632218 PMC6788410

[ref11] Chowdhury S. P. Hartmann A. Gao X. Borriss R. (2015). Biocontrol mechanism by root-associated *Bacillus amyloliquefaciens* FZB42-a review. Front. Microbiol. 6:780. doi: 10.3389/fmicb.2015.00780, PMID: 26284057 PMC4517070

[ref12] Dai Y. Wu X. Q. Wang Y. H. Zhu M. L. (2021). Biocontrol potential of *Bacillus pumilus* HR10 against *Sphaeropsis* shoot blight disease of pine. Biol. Control 152:104458. doi: 10.1016/j.biocontrol.2020.104458

[ref13] Dean R. Van Kan J. A. Pretorius Z. A. Hammond-Kosack K. E. Di Pietro A. Spanu P. D.. (2012). The top 10 fungal pathogens in molecular plant pathology. Mol. Plant Pathol. 13, 414–430. doi: 10.1111/j.1364-3703.2011.00783.x, PMID: 22471698 PMC6638784

[ref14] Díaz J.´. Have A. van Kan J. A. L. (2002). The role of ethylene and wound signaling in resistance of tomato to *Botrytis cinerea*. Plant Physiol. 129, 1341–1351. doi: 10.1104/pp.001453, PMID: 12114587 PMC166527

[ref15] Fontana D. C. de Paula S. Torres A. G. de Souza V. H. M. Pascholati S. F. Schmidt D.. (2021). Endophytic fungi: biological control and induced resistance to phytopathogens and abiotic stresses. Pathogens. 10:570. doi: 10.3390/pathogens10050570, PMID: 34066672 PMC8151296

[ref16] Gao P. Qin J. Zhou S. (2018). Inhibitory effect and possible mechanism of a pseudomonas strain QBA5 against gray mold on tomato leaves and fruits caused by *Botrytis cinerea*. PLoS One 13:e0190932. doi: 10.1371/journal.pone.0190932, PMID: 29320571 PMC5761960

[ref17] Grzegorczyk M. Cirvilleri G. (2017). Postharvest biocontrol ability of killer yeasts against *Monilinia fructigena* and *Monilinia fructicola* on stone fruit. Food Microbiol. 61, 93–101. doi: 10.1016/j.fm.2016.09.005 27697174

[ref18] Gui Y. J. Chen J. Y. Zhang D. D. Li N. Y. Li T. G. Zhang W. Q.. (2017). *Verticillium dahliae* manipulates plant immunity by glycoside hydrolase 12 proteins in conjunction with carbohydrate-binding module 1. Environ. Microbiol. 19, 1914–1932. doi: 10.1111/1462-2920.13695, PMID: 28205292

[ref19] Hamaoka K. Aoki Y. Suzuki S. (2021). Isolation and characterization of endophyte *Bacillus velezensis* KOF112 from grapevine shoot xylem as biological control agent for fungal diseases. Plants 10:1815. doi: 10.3390/plants10091815, PMID: 34579349 PMC8468208

[ref20] Hammond-Kosack K. E. Parker J. E. (2003). Deciphering plant pathogen communication: fresh perspectives for molecular resistance breeding. Curr. Opin. Biotechnol. 14, 177–193. doi: 10.1016/s0958-1669(03)00035-1 12732319

[ref21] Hernández A. F. Gil F. Lacasaña M. Rodríguez-Barranco M. Tsatsakis A. M. Requena M.. (2013). Pesticide exposure and genetic variation in xenobiotic-metabolizing enzymes interact to induce enzymes liver damage. Food Chem. Toxicol. 61, 144–151. doi: 10.1016/j.fct.2013.05.012, PMID: 23688862

[ref22] Javed K. Javed H. Qiu D. (2020). Biocontrol potential of purified elicitor protein PeBL1 extracted from *Brevibacillus laterosporus* strain *A60*and its capacity in the induction of defense process against cucumber aphid (*Myzus persicae*) in cucumber (*Cucumis sativus*). Biology 9:179. doi: 10.3390/biology9070179, PMID: 32708244 PMC7408455

[ref23] Ji X. B. Wang D. Liu Z. H. Li R. Song J. Zhang D. D.. (2021). Inhibitory efficacy of BvR001 against *Verticillium dahliae*. Plant Prot. 47, 40–47. doi: 10.16688/j.zwbh.2019568

[ref24] Ju R. Zhao Y. Li J. Jiang H. Liu P. Yang T.. (2014). Identification and evaluation of a potential biocontrol agent, *Bacillus subtilis*, against *fusarium* sp. in apple seedlings. Ann. Microbiol. 64, 377–383. doi: 10.1007/s13213-013-0672-3

[ref25] Kaewchai S. Soytong K. Hyde K. D. (2009). Mycofungicides and fungal biofertilizers. Fungal Divers. 38, 25–50. doi: 10.1002/yea.1704

[ref26] Kazerooni E. A. Maharachchikumbura S. S. Al-Sadi A. M. Kang S. M. Yun B. W. Lee I. J. (2021). Biocontrol potential of *Bacillus amyloliquefaciens* against *Botrytis pelargonii* and *Alternaria alternata* on *Capsicum annuum*. J. Fungi 7:472. doi: 10.3390/jof7060472 PMC8230671 34200967

[ref27] Khan N. Martínez-Hidalgo P. Ice T. A. Maymon M. Humm E. A. Nejat N.. (2018). Antifungal activity of *Bacillus* species against *fusarium* and analysis of the potential mechanisms used in biocontrol. Front. Microbiol. 9:2363. doi: 10.3389/fmicb.2018.02363, PMID: 30333816 PMC6176115

[ref28] Kim Y. S. Song J. G. Lee I. K. Yeo W. H. Yun B. S. (2013). *Bacillus* sp. BS061 suppresses powdery mildew and gray mold. Mycobiology. 41, 108–111. doi: 10.5941/MYCO.2013.41.2.108, PMID: 23874134 PMC3714439

[ref29] Leroux P. Elad Y. Williamson B. Tudzynski P. Delen N. (2004). “Chemical control of Botrytis and its resistance to chemical fungicides,” in Botrytis: Biology, Pathology and Control. Ed. Y. Elad, B. Williamson, P. Tudzynski and N. Delen (Dordrecht: Springer Dordrecht), 195–222.

[ref30] Liu C. Liu H. W. Wang B. Q. Zhao W. Y. Wang Y. N. Zhang L. P.. (2019). Isolation and antibacterial activity of *Bacillus amyloliquefaciens* BA-26 against *Botrytis cinerea*. Chin. J. Biotechnol. 35, 83–89. doi: 10.13560/l.1985.2019-0055

[ref31] Liu X. M. Ouyang C. B. Wang Q. X. Li Y. Yan D. D. Yang D. S. H.. (2017). Effects of oil extracts of *Eupatorium adenophorum* on *Phytophthora capsici* and other plant pathogenic fungi in vitro. Pestic. Biochem. Physiol. 140, 90–96. doi: 10.1016/j.pestbp.2017.06.012 28755701

[ref32] Livak K. J. Schmittgen T. D. (2001). Analysis of relative gene expression data using real-time quantitative PCR and the 2^–ΔΔCT^ method. Methods 25, 402–408. doi: 10.1007/978-1-4020-2626-3_12 11846609

[ref33] Moyes R. B. Reynolds J. Breakwell D. P. (2009). Differential staining of bacteria: gram stain. Curr. Protoc. Microbiol. Appendix 3:Appendix 3C. doi: 10.1002/9780471729259.mca03cs15 19885931

[ref34] Nalini S. Parthasarathi R. (2014). Production and characterization of rhamnolipids produced by *Serratia rubidaea* SNAU02 under solid-state fermentation and its application as biocontrol agent. Bioresour. Technol. 173, 231–238. doi: 10.1016/j.biortech.2014.09.051, PMID: 25305653

[ref35] Nan J. Zhang S. Jiang L. (2021). Antibacterial potential of *Bacillus amyloliquefaciens* GJ1 against citrus huanglongbing. Plants 10:261. doi: 10.3390/plants10020261, PMID: 33572917 PMC7910844

[ref36] Nifakos K. Tsalgatidou P. C. Thomloudi E. E. Skagia A. Kotopoulis D. Baira E.. (2021). Genomic analysis and secondary metabolites production of the endophytic *Bacillus velezensis* Bvel1: a biocontrol agent against *Botrytis cinerea* causing bunch rot in post-harvest table grapes. Plants. 10:1716. doi: 10.3390/plants10081716, PMID: 34451760 PMC8400388

[ref37] Nigris S. Baldan E. Tondello A. Zanella F. Vitulo N. Favaro G.. (2018). Biocontrol traits of *Bacillus licheniformis* GL174, a culturable endophyte of *Vitis vinifera* cv. *Glera*. BMC Microbiol. 18:133. doi: 10.1186/s12866-018-1306-5 30326838 PMC6192205

[ref38] Pandit M. A. Kumar J. Gulati S. Bhandari N. Mehta P. Katyal R.. (2022). Major biological control strategies for plant pathogens. Pathogens. 11:273. doi: 10.3390/pathogens11020273, PMID: 35215215 PMC8879208

[ref39] Pedras M. S. C. Hossain S. Snitynsky R. B. (2011). Detoxification of cruciferous phytoalexins in *Botrytis cinerea*: spontaneous dimerization of a camalexin metabolite. Phytochemistry 72, 199–206. doi: 10.1016/j.phytochem.2010.11.018, PMID: 21176925

[ref40] Pei D. Zhang Q. Zhu X. Zhang L. (2022). Biological control of *Verticillium* wilt and growth promotion in tomato by Rhizospheric soil-derived *Bacillus amyloliquefaciens* Oj-2.16. Pathogens. 12:37. doi: 10.3390/pathogens12010037, PMID: 36678385 PMC9865522

[ref41] Qiao J. Yu X. Liang X. Liu Y. Borriss R. (2017). Addition of plant-growth-promoting *Bacillus subtilis* PTS-394 on tomato rhizosphere has no durable impact on composition of root microbiome. BMC Microbiol. 17:131. doi: 10.1186/s12866-017-1039-x, PMID: 28583081 PMC5460418

[ref42] Rosslenbroich H. J. Stuebler D. (2000). *Botrytis cinerea*-history of chemical control and novel fungicides for its management. Crop Prot. 19, 557–561. doi: 10.1016/S0261-2194(00)00072-7

[ref43] Schwyn B. Neilands J. B. (1987). Universal chemical assay for the detection and determination of siderophores. Anal. Biochem. 160, 47–56. doi: 10.1016/0003-2697(87)90612-9, PMID: 2952030

[ref44] Simair A. A. Qureshi A. S. Khushk I. Ali C. H. Lashari S. Bhutto M. A.. (2017). Production and partial characterization of *α*-amylase enzyme from *Bacillus* sp. BCC 01-50 and potential applications. Biomed. Res. Int. 2017:9173040. doi: 10.1155/2017/9173040, PMID: 28168200 PMC5267059

[ref45] Sun X. Xu Z. Xie J. Hesselberg-Thomsen V. Tan T. Zheng D.. (2022). *Bacillus velezensis* stimulates resident rhizosphere *Pseudomonas stutzeri* for plant health through metabolic interactions. ISME J. 16, 774–787. doi: 10.1038/s41396-021-01125-3, PMID: 34593997 PMC8483172

[ref46] Thomma B. P. Penninckx I. A. Cammue B. P. Broekaert W. F. (2001). The complexity of disease signaling in *Arabidopsis*. Curr. Opin. Immunol. 13, 63–68. doi: 10.1016/S0952-7915(00)00183-7 11154919

[ref47] Varghese F. Bukhari A. B. Malhotra R. De A. (2014). IHC profiler: an open source plugin for the quantitative evaluation and automated scoring of immunohistochemistry images of human tissue samples. PLoS One 9:e96801. doi: 10.1371/journal.pone.0096801, PMID: 24802416 PMC4011881

[ref48] Villa-Rojas R. Sosa-Morales M. E. López-Malo A. Tang J. (2012). Thermal inactivation of *Botrytis cinerea* conidia in synthetic medium and strawberry puree. Int. J. Food Microbiol. 155, 269–272. doi: 10.1016/j.ijfoodmicro.2012.02.021, PMID: 22445202

[ref49] Wan M. G. Li G. Q. Zhang J. B. Jiang D. H. Huang H. C. (2008). Effect of volatile substances of *Streptomyces platensis* F-1 on control of plant fungal diseases. Biol. Control 46, 552–559. doi: 10.1016/j.biocontrol.2008.05.015

[ref50] Wang H. C. Li W. H. Wang M. S. Chen Q. Y. Feng Y. G. Shi J. X. (2011). First report of Botrytis cinerea causing gray Mold of tobacco in Guizhou Province of China. Plant Dis. 95:612. doi: 10.1094/PDIS-01-11-0064, PMID: 30731976

[ref51] Wang N. Liu M. Guo L. Yang X. Qiu D. A. (2016). Novel protein elicitor (PeBA1) from *Bacillus amyloliquefaciens* NC6 induces systemic resistance in tobacco. Int. J. Biol. Sci. 12, 757–767. doi: 10.7150/ijbs.14333, PMID: 27194952 PMC4870718

[ref52] Wang D. Luo W. Z. Zhang D. D. Li R. Kong Z. Q. Song J.. (2023). Insights into the biocontrol function of a *Burkholderia gladioli* strain against *Botrytis cinerea*. Microbiol Spectr. 11:e0480522. doi: 10.1128/spectrum.04805-22, PMID: 36861984 PMC10101029

[ref53] Wu H. S. Yang X. N. Fan J. Q. Miao W. G. Ling N. Xu Y. C.. (2009). Suppression of fusarium wilt of watermelon by a bio-organic fertilizer containing combinations of antagonistic microorganisms. Biol. Control 54, 287–300. doi: 10.1007/s10526-008-9168-7

[ref54] Yuan Y. Feng H. Wang L. Li Z. Shi Y. Zhao L.. (2017). Potential of endophytic fungi isolated from cotton roots for biological control against Verticillium wilt disease. PLoS One 12:e0170557. doi: 10.1371/journal.pone.0170557, PMID: 28107448 PMC5249208

[ref55] Zhang Y. B. Liu X. Y. Wang Y. F. Jiang P. P. Quek S. Y. (2016). Antibacterial activity and mechanism of cinnamon essential oil against *Escherichia coli* and *Staphylococcus aureus*. Food Control 59, 282–289. doi: 10.1016/j.foodcont.2015.05.032

[ref56] Zheng Y. Wang X. Liu S. Zhang K. Cai Z. Chen X.. (2018). The endochitinase of *Clonostachysrosea* expression in *Bacillus amyloliquefaciens* enhances the *Botrytis cinerea* resistance of tomato. Int. J. Mol. Sci. 19:2221. doi: 10.3390/ijms19082221, PMID: 30061502 PMC6121428

[ref57] Zhou Q. Fu M. Xu M. Chen X. Qiu J. Wang F.. (2020). Application of antagonist *Bacillus amyloliquefaciens* NCPSJ7 against *Botrytis cinerea* in postharvest red globe grapes. Food Sci. Nutr. 8, 1499–1508. doi: 10.1002/fsn3.1434, PMID: 32180959 PMC7063376

