# Supplementary material for: Unlocking antagonistic potential of Bacillus amyloliquefaciens KRS005 to control gray mold
Source: Front Microbiol. 2023 Jun 2;14:1189354. doi: 10.3389/fmicb.2023.1189354 (PMC10272387; doi:10.3389/fmicb.2023.1189354)
Supplement: Supplementary file 1 [file Table_1.DOCX]

Supplementary Material

Unlocking Antagonistic Potential of *Bacillus* *amylotiaquefaciens* KRS005 to Control Grey Mold Infection

**Hong-Yue Qi^1,2^†, Dan Wang^2^†, Xiao-Jun Zhang^1^*****, Jie-Yin Chen^2,5^***

*** Correspondence:** Jie-Yin Chen: E-mail: [chenjieyin@caas.cn](mailto:chenjieyin@caas.cn);

Xiao-Jun Zhang: E-mail: [swxzxj@126.com](mailto:swxzxj@126.com);

**Table S1 Oligonucleotide primers used in this study.**

| **Primer name** | **Primer sequence (5′-3′)** |
| --- | --- |
| *gyrB, gyrA, ropB gene cloning* |  |
| *gyrB*-UP1R | GAAGTCATCATGACCGTTCTGCA(C/T)GC(A/G/C/T)GG(A/G/C/T)AA(A/G)TT(C/T)GA |
| *gyrB*-UP2F | AGCAGGGTACGGATGTGCGAGCC(A/GTC(A/G/C/T)AC(A/G)TC(A/G/C/T)GC(A/G)TC(A/G/C/T)GTCAT |
| *gyrA*-L100F | AAATCTGCCCGTATCGTCG |
| *gyrA*-R836R | GCGTCACGGCGRATCTCAA |
| *rpoB*-2292F | AGGTCAACTAGTTCAGTATGGAC |
| *rpoB*-3354R | AAGAACCGTAACCGGCAACTT |
| *resistance genes expression* |  |
| *NbHSR203-F* | CCGCCTTCCCTCAACTCAAC |
| *NbHSR203-R* | GCACAACCAAGACGTACTGAG |
| *NbHIN1-F* | AGGTGTTTGCTATGGAATGC |
| *NbHIN1-R* | TCTGTACCCACCATCTTGC |
| *NbPR1a-F* | GGGCCAATCTTGGAGCATTA |
| *NbPR1a-R* | CAGTCTCCAGTCTCACAATTACC |
| *NbPR2-F* | GCTAGATTAGCCCTTGCTTCA |
| *NbPR2-R* | ATTCCTTCCTGCTGTCAGATTAG |
| *NbPR4-F* | GGCCAAGATTCCTGTGGTAGAT |
| *NbPR4-R* | CACTGTTGTTTGAGTTCCTGTTCCT |
| *NbLOX-F* | AAAACCTATGCCTCAAGAAC |
| *NbLOX-R* | ACTGCTGCATAGGCTTTGG |
| *NbRbohA-F* | GCAAGGAGTACTGTGTCCATTA |
| *NbRbohA-R* | CCTTGCACCTCAAACTCCTAT |
| *NbRbohB-F* | ATCCTCGGAGTGATTGCATTAG |
| *NbRbohB-R* | TGTTGTTTGTGGTGGACAAATC |
| *Fungal biomass detection* |  |
| *NbEF-F* | AGGATACAACCCTGACAAGA |
| *NbEF-R* | GTGGGACCAAAAGTAACAAC |
| *BcActin-F* | TCCAAGCGTGGTATTCTTACCC |
| *BcActin-R* | TGGTGCTACACGAAGTTCGTTG |
